# Pre-metastatic Niche Formation in Different Organs Induced by Tumor Extracellular Vesicles

**DOI:** 10.3389/fcell.2021.733627

**Published:** 2021-09-20

**Authors:** Qi Dong, Xue Liu, Ke Cheng, Jiahao Sheng, Jing Kong, Tingjiao Liu

**Affiliations:** ^1^Department of Basic Science of Stomatology, Shanghai Stomatological Hospital, Fudan University, Shanghai, China; ^2^Shanghai Key Laboratory of Craniomaxillofacial Development and Diseases, Fudan University, Shanghai, China; ^3^Department of Oral Pathology, School of Stomatology, Dalian Medical University, Dalian, China

**Keywords:** pre-metastatic niche (PMN), extracellular vesicles (EVs), vascular permeability, extracellular matrix (ECM), bone marrow-derived cells (BMDCs), immunosuppression

## Abstract

Primary tumors selectively modify the microenvironment of distant organs such as the lung, liver, brain, bone marrow, and lymph nodes to facilitate metastasis. This supportive metastatic microenvironment in distant organs was termed the pre-metastatic niche (PMN) that is characterized by increased vascular permeability, extracellular matrix remodeling, bone marrow-derived cells recruitment, angiogenesis, and immunosuppression. Extracellular vesicles (EVs) are a group of cell-derived membranous structures that carry various functional molecules. EVs play a critical role in PMN formation by delivering their cargos to recipient cells in target organs. We provide an overview of the characteristics of the PMN in different organs promoted by cancer EVs and the underlying mechanisms in this review.

## Introduction

Primary tumors selectively modify the microenvironment of distant organs before metastasis (Hiratsuka et al., 2002; Kaplan et al., 2005). This supportive metastatic microenvironment in distant organs was first termed the pre-metastatic niche (PMN) by Kaplan et al. (2005). In the last decade, PMN induced by various cancers has been identified in the lung (Hiratsuka et al., 2002; Hoshino et al., 2015; Liu et al., 2016; Tyagi et al., 2021), liver (Hoshino et al., 2015; Zhang and Wang, 2015; Houg and Bijlsma, 2018; Sun et al., 2021), brain (You et al., 2020), bone (Kaplan et al., 2007; Xu et al., 2017), and other organs. The PMN is characterized by increased vascular permeability (Gupta et al., 2007; Huang et al., 2009; Zhou et al., 2014), a modified extracellular matrix (ECM) (Aguado et al., 2016; Kim et al., 2019; Mohan et al., 2020), recruited bone marrow-derived cells (BMDCs) (Kitamura et al., 2015; Wang et al., 2019), and immunosuppression (Chen et al., 2011; Tacke et al., 2012; Giles et al., 2016) in the future metastatic organs.

Extracellular vesicles (EVs) are a heterogeneous group of nano-sized membranous structures that are released by almost all cells into extracellular spaces and have many different physiological and pathophysiological functions (Raposo and Stoorvogel, 2013; Colombo et al., 2014). Different EV types, including exosomes, microvesicles, apoptotic bodies, oncosomes, and megasomes have been characterized on the basis of their biogenesis pathways and sizes. Among them, exosomes and microvesicles are the most intensively studied types. Exosomes have an endocytic origin and form by the fusion between multivesicular bodies and the plasma membrane, whereas microvesicles are generated by plasma membrane shedding (van Niel et al., 2018). However, it is difficult to classify EVs according to their biogenic origin once they are secreted into the extracellular space. Therefore, size-based or density-based nomenclature is recommended by the International Society of Extracellular Vesicles (Thery et al., 2018). EVs contain various functional molecules (proteins, mRNAs, miRNAs, long non-coding RNAs, and double stranded DNA, etc.) that can be trafficked between cells as a means of intercellular communication at both paracrine and systemic levels (Valadi et al., 2007; Balaj et al., 2011; Choi et al., 2013; Thakur et al., 2014). EV cargos are protected by the membrane during the delivery process, which is critical for the communication between primary tumors and distant organs. EVs can be internalized by recipient cells via different mechanisms, including phagocytosis, macropinocytosis, endocytosis, and direct membrane fusion (van Niel et al., 2018). In addition, ligands on EV membrane can interact with receptors on the recipient cell surface and elicit biological responses directly.

In this mini-review, we provide an overview of the characteristics of the PMN in different organs promoted by cancer EVs. The terms, exosomes, microvesicles, or EVs were used to describe their roles in PMN formation to ensure consistency with the original articles.

## Characteristics of the Pre-Metastatic Niche

**FIGURE 1:**
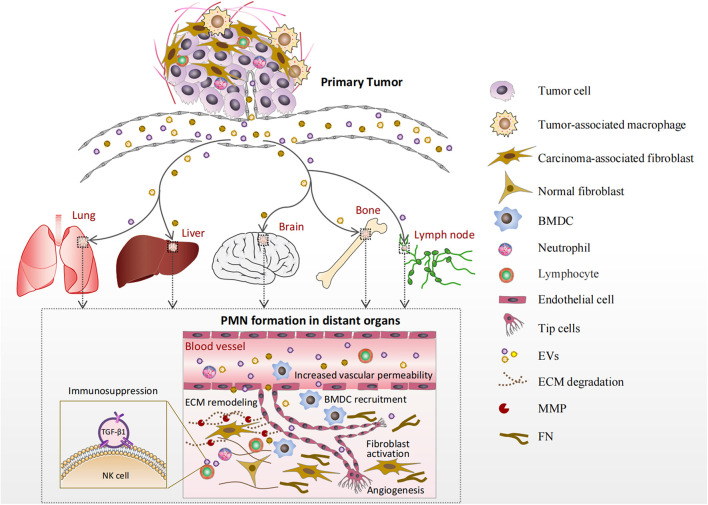
Formation of the pre-metastatic niche (PMN) in various organs. Extracellular vesicles (EVs) produced by tumor and stromal cells enter the circulation and arrive at distant organs such as the lung, liver, brain, bone marrow, and lymph nodes (LNs). Increased vascular permeability is an early event in PMN formation. Then, EVs cause extracellular matrix (ECM) remodeling principally by activating resident normal fibroblasts. The activated fibroblasts deposit new ECM components, such as FN, and produce matrix metalloproteinases (MMPs). Bone marrow-derived cells (BMDCs) are recruited into the target organs by EVs and involved in angiogenesis and/or immune responses. Furthermore, EVs can deliver their cargos to endothelial cells or immunocytes directly in the PMN to promote angiogenesis or create an immunosuppressive microenvironment.

The characteristics of the PMN formed in various organs by EVs are summarized in Figure 1 and Table 1. The lung is the most commonly involved organ, followed by the liver, bone, brain, and lymph nodes (LNs). As shown in Figure 1, EVs produced by tumor and stromal cells enter the circulation and arrive at distant organs, where they trigger a sequence of local changes including increased vascular permeability, ECM remodeling, BMDC recruitment, angiogenesis, and immunosuppression.

**TABLE 1:** Pre-metastatic niche (PMN) formation promoted by extracellular vesicles (EVs).

Organs	Tumor	PMN-promoting EVs	PMN characteristics	References
Lung	Breast cancer	Exosomes from EO771 cells	BMDC recruitment and immunosuppression	Wen et al., 2016
Lung	Breast cancer	EV miR-122	Reprograms glucose metabolism	Fong et al., 2015
Lung	Breast cancer	miR-105-rich EVs	Vascular permeability	Zhou et al., 2014
Lung	Breast Cancer	Exosome-associated Annexin II	Angiogenesis	Maji et al., 2017
Lung	Melanoma	Pigment epithelium-derived factor-positive exosomes	Immune cells recruitment	Plebanek et al., 2017
Lung	Melanoma	Melanoma-derived exosomes	BMDC recruitment; Vascular permeability	Peinado et al., 2012
Lung	Melanoma	EVs from insulin-like growth factor 2 mRNA-binding protein 1 (IGF2BP1)-overexpressed/knockdown melanoma cells	ECM remodeling (fibronectin deposition); Recruit CD45 + cells in the lung	Ghoshal et al., 2019
Lung	Hepatocellular carcinoma	Nidogen 1 (NID1) in metastatic HCC cell-derived EVs	Angiogenesis	Mao et al., 2020
Lung	High-metastatic hepatocellular carcinoma	miR-1247-3p positive exosomes	Inflammatory microenvironment	Fang et al., 2018
Lung	Colorectal cancer	Exosomal miR-25-3p	Vascular permeability; Angiogenesis	Zeng et al., 2018
Lung	Colorectal cancer	Integrin beta-like 1-rich-EVs	Inflammatory microenvironment	Ji et al., 2020
Lung	Pancreatic ductal adenocarcinomas	Exosomal Podocalyxin	ECM remodeling	Novo et al., 2018
Lung	Osteosarcoma	Osteosarcoma (143-B) cells-derived EVs-associated TGFβ1	Fibroblast activation	Mazumdar et al., 2020b
Lung	Osteosarcoma	Highly metastatic 143-B osteosarcoma cell-derived EVs	CD11b + myeloid cells recruitment	Mazumdar et al., 2020a
Lung	Prostate cancer	Exosomes from human prostate cancer (PCa) PC3 cells under hypoxic conditions	ECM remodeling; BMDC recruitment	Deep et al., 2020
Lung	Nasopharyngeal carcinoma	EV packaged latent membrane protein 1	Fibroblast activation	Wu et al., 2020
Lung	SACC	Epiregulin-positive exosomes	Vascular permeability; Macrophage recruitment	Yang et al., 2017
Lung	Human renal cell carcinoma	MVs derived from CD105-positive cancer stem cells	Angiogenesis	Grange et al., 2011
Lung	Non-small cell lung carcinoma (NSCLC)	Exosomal RNAs (small nuclear RNAs snRNAs)	Immunosuppression	Liu et al., 2016
Lung	Metastatic rat adenocarcinoma	Exosomes from the metastatic rat adenocarcinoma BSp73ASML (ASML)	ECM remodeling; Immunosuppression	Rana et al., 2013
Liver	Breast Cancer	Breast cancer derived-EVs-associated nucleoside diphosphate kinase A and B(NDPK)	Vascular permeability	Duan et al., 2021
Liver	Pancreatic cancer	Exosomes from the highly metastatic pancreatic cancer cell line (Panc02-H7 EXO)	BMDC recruitment	Yu et al., 2017
Liver	Pancreatic Cancer	EV-associated TGF-β1	Immunosuppression	Zhao et al., 2019
Liver	Pancreatic ductal adenocarcinomas	PDAC-derived exosomes-associated macrophage migration inhibitory factor (MIF)	ECM remodeling; BMDC recruitment	Costa-Silva et al., 2015
Liver	Gastric cancer	EGFR-containing EVs	ECM remodeling	Zhang et al., 2017
Liver	Colorectal cancer	Exosomal miR-25-3p	Vascular permeability; Angiogenesis	Zeng et al., 2018
Liver	Colorectal cancer	Integrin beta-like 1-rich-EVs	Inflammatory microenvironment	Ji et al., 2020
Bone	Lung cancer	miR-192-enriched- exosome-like vesicles (ELV)	Angiogenesis	Valencia et al., 2014
Bone	Prostate cancer	Phospholipase D (PLD) isoforms PLD2-riched exosomes	Shift the bone balance in favor of osteoblasts	Borel et al., 2020
Bone	Prostate cancer	Enzalutamide resistant (EnzR) CWR-R1 cells derived EVs (EnzR EVs)	BMDC recruitment	Henrich et al., 2020
Brain	Breast cancer and melanoma	EVs derived from brain metastases cancer cells (Br-EVs)	Blood–brain barrier (BBB) permeability	Busatto et al., 2020
Brain	Glioblastoma multiforme	EV-associated VEGF-A	Angiogenesis	Treps et al., 2017
LNs	Metastatic rat adenocarcinoma	Exosomes from the metastatic rat adenocarcinoma BSp73ASML (ASML)	ECM remodeling; Immunosuppression	Rana et al., 2013
LNs	Melanoma	Melanoma-derived exosomes	ECM remodeling; Angiogenesis	Hood et al., 2011

### Increased Vascular Permeability

Increased vascular permeability is an early event in PMN formation (Huang et al., 2009; Araldi et al., 2012). Vascular destabilization in the PMN promotes the extravasation of tumor cells and facilitates metastasis. Both EV-associated miRNAs and proteins contribute to vascular destabilization by destroying adhesion molecules between endothelial cells. Exosomal miR-25-3p derived from human colorectal cancer cells promotes vascular permeability in mouse models by regulating the expression of the tight junction proteins zonula occludens-1 (ZO-1), occludin, and claudin-5 in endothelial cells, thereby promoting cancer metastasis in the liver and lungs of mice (Zeng et al., 2018). In breast cancer, metastatic cancer cells secrete miR-105-rich exosomes that regulate vascular permeability and promote tumor metastasis by downregulating ZO-1 expression (Zhou et al., 2014). Melanoma-derived exosomes upregulate tumor necrosis factor-α expression in the lung, disrupting endothelial cell–cell junctions and increasing vascular permeability (Peinado et al., 2012). Vascular endothelial growth factor-A (VEGF-A) is carried by EVs derived from glioblastoma stem-like cells, increasing vascular permeability *in vivo* and the angiogenic potential of human brain endothelial cells (Treps et al., 2017). In addition, nucleoside diphosphate kinase B enriched in EVs derived from triple negative breast cancer cells (MDA-MB-231) enhances pulmonary blood vessel leakage and experimental lung metastasis (Duan et al., 2021).

### Extracellular Matrix Remodeling

Extracellular matrix remodeling, a key event in PMN formation, is characterized by the deposition of new ECM components and the expression of enzymes related to ECM modification. The remodeled ECM provides substrates for incoming cancer cells and increases matrix stiffness, which affects the properties of cancer cells (Erler et al., 2009; Cox et al., 2013; Wu et al., 2021). Several ECM components are involved in PMN formation, including fibronectin (Murgai et al., 2017), tenascin-C (Urooj et al., 2020), periostin (Malanchi et al., 2011; Fukuda et al., 2015; Wang et al., 2016), and versican (Kim et al., 2009; Gao et al., 2012). Fibronectin is reported to be upregulated in the livers of mice treated with exosomes from highly metastatic pancreatic cancer cells (Yu et al., 2017). Macrophage migration inhibitory factor (MIF) is highly expressed in pancreatic cancer cell-derived exosomes. Uptake of exosomal MIF by liver Kupffer cells causes transforming growth factor-β (TGF-β) secretion, leading to the activation of hepatic stellate cells. Fibronectin production by the activated hepatic stellate cells promotes the infiltration of bone marrow-derived macrophages and neutrophils in the liver, leading to the formation of the PMN (Costa-Silva et al., 2015). Insulin-like growth factor 2 mRNA-binding protein 1 is rich in melanoma cell EVs and promotes PMN formation in the lungs through the deposition of fibronectin and accumulation of CD45^+^ cells (Ghoshal et al., 2019). Exosomes from mutant p53-expressing pancreatic ductal adenocarcinoma cells affect the deposition and remodeling of the ECM by fibroblasts to generate a microenvironment highly supportive of tumor cell migration and invasion (Novo et al., 2018). Proteolytic enzymes play a critical role in ECM remodeling during PMN formation. Matrix metalloproteinases (MMPs) are a family of zinc-dependent endopeptidases that target ECM proteins, and MMP induction is one of the hallmarks of PMN formation. Deep et al. (2020) found that exosomes secreted by prostate cancer cells under hypoxia increase MMP activity and upregulate the expression of MMP-2, MMP-9, fibronectin, and collagen IV in the lung, liver, kidney, and spleen. A growing number of EV-associated miRNA have been involved in the regulation of ECM in regional sites. EV-associated miR-494 and miR-542-3p in metastatic rat adenocarcinoma cells regulate tumor-draining LNs and lung tissue by upregulating MMPs to form the PMN (Rana et al., 2013).

Stromal ECM proteins are mainly produced by fibroblasts. ECM remodeling by activated fibroblasts in the PMN has been reported by several groups. Wu et al. (2020) demonstrated that EVs from nasopharyngeal carcinoma package latent membrane protein 1 and activate the conversion of normal fibroblasts into carcinoma-associated fibroblasts (CAFs), thus, increasing the levels of typical PMN biomarkers, including fibronectin, S100A8, and VEGFR1 in lung and liver tissues. Ji et al. (2020) reported that colorectal cancer cells release integrin beta-like 1 (ITGBL1)-rich EVs that activate fibroblasts through the EVs-ITGBL1-CAFs-TNFAIP3-NF-κB axis in the liver and lung; the activated fibroblasts promote PMN formation by secreting pro-inflammatory factors. Fang et al. (2018) found that the highly metastatic hepatocellular carcinoma cell-derived exosomal miR-1247-3p induces the activation of normal fibroblasts into CAFs, which secrete inflammatory cytokines such as IL-6 and IL-8 to promote PMN formation in the lung, thus, promoting lung metastasis of liver cancer. Mazumdar et al. (2020b) provided strong evidence that osteosarcoma cell-derived EVs can activate fibroblasts into CAF phenotypes in the lung PMN through EV-associated TGF-β1 and SMAD2 pathway activation.

### Bone Marrow-Derived Cell Recruitment

One mechanism by which tumor factors promote PMN formation is by mobilizing BMDCs to establish a suitable environment in specific secondary organs. The proto-oncoprotein MET is a receptor tyrosine kinase involved in cancer cell growth and invasion. Exosomes from highly metastatic melanomas reprogram BMDCs toward a pro-vasculogenic phenotype by transferring EV-associated MET, and thus, increasing the metastatic behavior of primary tumors (Peinado et al., 2012). Osteosarcoma-derived EVs can increase CD11b^+^ myeloid cell infiltration in the lungs (Mazumdar et al., 2020a). In pancreatic cancer, highly metastatic pancreatic cancer cell-derived exosomes recruit CD11b^+^ and CD45^+^ hematopoietic progenitor cells at the PMN (Yu et al., 2017). An opposite example of tumor EVs promoting PMN formation is the inhibition of lung metastasis in melanoma cells with low metastatic potential (Plebanek et al., 2017). These low-metastatic tumor cell-derived exosomes can amplify Ly6C^low^ patrolling monocytes in the bone marrow and trigger a wide range of innate immune responses. The pigment epithelial-derived factor on the external surface of exosomes plays a critical role in the process.

### Angiogenesis

Angiogenesis is a prominent characteristic of the PMN. It was demonstrated that treating immunodeficient mice with epiregulin-enriched exosomes derived from salivary adenoid cystic carcinoma greatly enhanced tumor metastasis to the lung. Epiregulin-enriched exosomes upregulate the expression of VEGF, FGF-2, IL-8, and VEGFR1 in lung vascular endothelial cells, thus, contributing to angiogenesis (Yang et al., 2017). Human renal cancer stem cells promote angiogenesis and the formation of a PMN in the lung; the process involves the induction of pro-angiogenic mRNAs and miRNAs by CD105^+^ microvesicles in the whole organ (Grange et al., 2011). Nidogen 1 in metastatic hepatocellular carcinoma cell-derived EVs is reported to promote PMN formation in the lung by enhancing angiogenesis and pulmonary endothelial permeability to facilitate colonization of tumor cells. EV-associated nidogen 1 activates fibroblasts to secrete tumor necrosis factor receptor 1, thereby facilitating lung colonization of tumor cells (Mao et al., 2020). Annexin II is one of the most highly expressed proteins in exosomes. Maji et al. (2017) showed that exosomal Annexin II generates a PMN to facilitate breast cancer metastasis in distant organs by promoting tissue plasminogen activator-dependent angiogenesis.

### Immunosuppression

The PMN is an immunosuppressive microenvironment comprising T cells, natural killer (NK) cells, neutrophils, monocytes, and macrophages (Seubert et al., 2015; Patel et al., 2018). Wen et al. (2016) reported that exosomes derived from highly metastatic murine breast cancer cells are distributed predominantly to the lungs of mice, where they suppress T-cell proliferation and inhibit NK cell cytotoxicity, likely suppressing the anticancer immune response in premetastatic organs. EVs isolated from pancreatic cancer induce a dysfunctional phenotype in NK cells, which contributes to an immunosuppressive microenvironment in the liver, and ultimately results in PMN formation. The study provided evidence that pancreatic cancer-derived EVs induce Smad2/3 phosphorylation and downregulate NKG2D in NK cells by delivering TGF-β1 to NK cells, which contributes to PMN formation in liver (Zhao et al., 2019). It is demonstrated that lung alveolar epithelial cells are stimulated by tumor exosomal RNAs via Toll-like receptor 3, which triggers neutrophil recruitment and lung metastatic niche formation (Liu et al., 2016). EVs secreted by brain metastases cells cause low-density lipoprotein aggregation, which accelerates EVs uptake by monocytes and macrophages. These monocytes and macrophages secrete immunosuppressive factors such as interleukin 10, chemokine ligand 2, and TGF-β, which contribute to PMN formation (Busatto et al., 2020). Maus et al. (2019) investigated lymphatic EVs of melanoma patients and showed that EVs traffic from the primary tumor microenvironment to the sentinel LNs and regulate the immune microenvironment in LNs, thereby promoting PMN formation.

### Others

Altered glucose metabolism, a hallmark of cancer, is characterized by increased glycolysis and glucose uptake. Fong et al. (2015) demonstrated that EV-associated microRNA-122 secreted by breast cancer cells can be transferred to normal cells (lung fibroblasts, brain astrocytes, and neurons) in the PMN, leading to reduced glucose uptake in these cells. Thereby, the niche accommodates a massive energy for cancer cell metastatic growth by suppressing the nutrient utilization in other cell types.

## Pre-Metastatic Niche Formation in Different Organs

### Lung

Our understanding of PMN biology is mostly based on studies of lung metastasis. Tumor EVs can target lung endothelial cells and cause vascular leakage and angiogenesis at pre-metastatic sites (Grange et al., 2011; Peinado et al., 2012; Zhou et al., 2014; Maji et al., 2017; Yang et al., 2017; Zeng et al., 2018; Mao et al., 2020). Lung fibroblasts are another common target of tumor EVs at PMN sites. Tumor EVs induce lung fibroblast reprogramming, by which fibroblasts are activated and differentiate into myofibroblast/CAF phenotypes, resulting in ECM remodeling, angiogenesis, secretion of pro-inflammatory cytokines, and immunosuppression (Fang et al., 2018; Novo et al., 2018; Ji et al., 2020; Mazumdar et al., 2020b; Wu et al., 2020). Alveolar epithelial cells released from tumor EVs secrete chemokines and neutrophils in the lungs to promote the formation of the microenvironment before lung metastasis (Liu et al., 2016). In addition, BMDCs (CD45^+^) from tumor exosomes in the lung inhibit the proliferation of T cells and the cytotoxicity of NK cells to form an immunosuppressive microenvironment (Wen et al., 2016). Overall, EVs target lung endothelial cells, fibroblasts, and alveolar epithelial cells to construct an inflammatory and immunosuppressive niche for the colonization of circulating tumor cells.

### Liver

Tumor EVs can be internalized by Kupffer cells (F4/80 positive) and hepatic stellate cells (alpha smooth muscle actin and desmin positive) in the liver and activate downstream signaling pathways. Costa-Silva et al. showed that macrophage MIF is highly expressed in pancreatic cancer-derived exosomes. Uptake of these exosomes by liver Kupffer cells causes TGF-β secretion, leading to activation of hepatic stellate cells. Fibronectin production by activated hepatic stellate cells promotes the infiltration of bone marrow-derived macrophages and neutrophils in the liver, leading to the formation of the PMN (Costa-Silva et al., 2015). In gastric cancer cells, epidermal growth factor receptor-containing exosomes target Kupffer cells and hepatic stellate cells to favor the development of a liver-like microenvironment, thereby promoting liver-specific metastasis (Zhang et al., 2017). In addition, cancer-derived EVs may target NK cells in the liver. Zhao et al. (2019) demonstrated that pancreatic cancer-derived EVs induce a dysfunctional phenotype of NK cells, which contributes to an immunosuppressive microenvironment in the liver, and ultimately results in PMN formation. These studies indicate that EVs establish a fibrotic and immunosuppressive liver PMN mainly through Kupffer cells and hepatic stellate cells, but not hepatocytes. It suggests that targeting circulation EVs and/or the fibrotic niche may provide an early prevention and therapeutic intervention for liver metastasis.

### Brain

Disruption of the blood-brain barrier is related to metastatic initiation and progression. Treps et al. (2017) reported that VEGF-A is carried by EVs derived from glioblastoma stem-like cells, and VEGF-A-enriched EVs promote PMN formation in the brain by targeting endothelial cells and increasing vascular permeability. In the same study, they demonstrated that EV-associated VEGF-A exerts pro-angiogenic activity on brain endothelial cells to stimulate angiogenesis. In addition, EVs may target non-endothelial cells in the brain to promote cancer brain metastasis. Busatto et al. revealed that brain metastasis cell-derived EVs interact with low-density lipoprotein and accelerate EV uptake by monocytes. These monocytes are key components in the brain niche and secrete immunosuppressive factors, such as interleukin 10, chemokine ligand 2, and TGF-β, which contribute to PMN formation (Busatto et al., 2020).

### Bone Marrow

Bone marrow myeloid cells and osteoblasts may contribute to PMN formation in the bone marrow. Henrich et al. characterized EV-mediated communication between prostate cancer cells and bone marrow myeloid cells, and demonstrated that cholesterol homeostasis in bone marrow myeloid cells regulates pro-metastatic EV signaling and metastasis. The phospholipase D (PLD) isoforms PLD1/2 regulate tumor progression and metastasis by catalyzing the hydrolysis of phosphatidylcholine to yield phosphatidic acid (Henrich et al., 2020). Borel et al. (2020) demonstrated that PLD2 is present in EVs of prostate cancer cells and activates proliferation and differentiation of osteoblasts by stimulating ERK 1/2 phosphorylation, leading to the formation of a microenvironment before bone metastasis. By contrast, Valencia et al. reported that lung cancer cells release miR-192-enriched EVs, which target endothelial cells and show antimetastatic activity *in vivo*. EV-associated miR-192 is internalized by endothelial cells and inhibits the expression of proangiogenic factors including IL-8, ICAM, and CXCL1 to reduce metastatic colonization (Valencia et al., 2014). Bone marrow is a complex microenvironment and contains multiple cell types such as osteoblasts, osteoclasts, myeloid cells, fibroblasts, macrophages, adipocytes, and endothelial cells. More studies are required to elucidate how these cells cooperate in PMN formation in the bone marrow.

### Lymph Nodes

Metastasis to LNs is common in various cancers. Melanoma-derived exosomes home to sentinel LNs and can convert a remote LN into a PMN before tumor cell colonization by inducing the expression of factors responsible for cell recruitment, matrix remodeling, and angiogenesis (Hood et al., 2011). Maus et al. (2019) investigated lymphatic EVs of melanoma patients and showed that EVs traffic from the primary tumor microenvironment to the sentinel LNs, where they regulate the immune microenvironment, thereby initiating PMN formation. However, these studies did not identify the definite target cells of tumor EVs in LNs.

## Clinical Implications and Future Perspectives

Studies show that various cancers can promote PMN formation in the lung, liver, brain, bone, and LNs by targeting endothelial cells, fibroblasts, alveolar epithelial cells, Kupffer cells, hepatic stellate cells, monocytes, and macrophages in these organs. Tumor EVs play a critical role in the communication between the primary tumor and distant organs by precisely delivering tumor products to target cells. Thus, tumor EVs in circulation should be a potential target of liquid biopsy to predict metastasis. Blocking the delivery of tumor EVs to target cells may prevent cancer metastasis. However, the biomarkers of tumor EVs that promote PMN formation have not been fully identified, and the mechanisms underlying the uptake of tumor EVs by target cells remain unclear. Except tumor cell-derived EVs, our group demonstrated that EVs secreted by CAFs can promote PMN formation in the lung by activating lung fibroblasts (Kong et al., 2019). Further study is necessary to determine whether EVs from other stromal cells in the primary tumor can induce PMN formation in more distant organs. Potential future studies in the field may focus on the following: (1) the different roles of EVs secreted by tumor and stromal cells in PMN formation; (2) the biomarkers of tumor or stromal EVs that promote PMN formation in various cancers; (3) the mechanisms by which recipient cells take up tumor or stromal EVs. The identification of PMN characteristics and a better understanding of the roles of EVs in PMN formation in various organs may help prevent cancer metastasis at an early stage.

## Author Contributions

QD, XL, KC, JS, and JK collected the literatures. TL and QD prepared the manuscript. All authors contributed to the article and approved the submitted version.

## Conflict of Interest

The authors declare that the research was conducted in the absence of any commercial or financial relationships that could be construed as a potential conflict of interest.

## Publisher’s Note

All claims expressed in this article are solely those of the authors and do not necessarily represent those of their affiliated organizations, or those of the publisher, the editors and the reviewers. Any product that may be evaluated in this article, or claim that may be made by its manufacturer, is not guaranteed or endorsed by the publisher.
